# Identification and replication of sex-dimorphic protein quantitative trait loci across multiple ancestries and their associations with diseases

**DOI:** 10.1038/s41598-025-10031-z

**Published:** 2025-08-28

**Authors:** Youngjune Bhak, Vasilis Raptis, Yunye He, Tomoko Nakanishi, Erin Macdonald-Dunlop, Yoji Sagiya, Takayuki Morisaki, Koichi Matsuda, BioBank Japan Project, The Japan COVID-19 Task Force, Ho Namkoong, Ryuya Edahiro, Tomomi Takano, Hiroshi Nishihara, Yuya Shirai, Kyuto Sonehara, Hiromu Tanaka, Shuhei Azekawa, Yohei Mikami, Ho Lee, Takanori Hasegawa, Koji Okudela, Daisuke Okuzaki, Daisuke Motooka, Masahiro Kanai, Tatsuhiko Naito, Kenichi Yamamoto, Qingbo S. Wang, Ryunosuke Saiki, Rino Ishihara, Yuta Matsubara, Junko Hamamoto, Hiroyuki Hayashi, Yukihiro Yoshimura, Natsuo Tachikawa, Emmy Yanagita, Takayoshi Hyugaji, Eigo Shimizu, Kotoe Katayama, Yasuhiro Kato, Takayoshi Morita, Kazuhisa Takahashi, Norihiro Harada, Toshio Naito, Makoto Hiki, Yasushi Matsushita, Haruhi Takagi, Ryousuke Aoki, Ai Nakamura, Sonoko Harada, Hitoshi Sasano, Hiroki Kabata, Katsunori Masaki, Hirofumi Kamata, Shinnosuke Ikemura, Shotaro Chubachi, Satoshi Okamori, Hideki Terai, Atsuho Morita, Takanori Asakura, Junichi Sasaki, Hiroshi Morisaki, Yoshifumi Uwamino, Kosaku Nanki, Sho Uchida, Shunsuke Uno, Tomoyasu Nishimura, Takashi Ishiguro, Taisuke Isono, Shun Shibata, Yuma Matsui, Chiaki Hosoda, Kenji Takano, Takashi Nishida, Yoichi Kobayashi, Yotaro Takaku, Noboru Takayanagi, Soichiro Ueda, Ai Tada, Masayoshi Miyawaki, Masaomi Yamamoto, Eriko Yoshida, Reina Hayashi, Tomoki Nagasaka, Sawako Arai, Yutaro Kaneko, Kana Sasaki, Etsuko Tagaya, Masatoshi Kawana, Ken Arimura, Kunihiko Takahashi, Tatsuhiko Anzai, Satoshi Ito, Akifumi Endo, Yuji Uchimura, Yasunari Miyazaki, Takayuki Honda, Tomoya Tateishi, Shuji Tohda, Naoya Ichimura, Kazunari Sonobe, Chihiro Tani Sassa, Jun Nakajima, Yasushi Nakano, Yukiko Nakajima, Ryusuke Anan, Ryosuke Arai, Yuko Kurihara, Yuko Harada, Kazumi Nishio, Tetsuya Ueda, Masanori Azuma, Ryuichi Saito, Toshikatsu Sado, Yoshimune Miyazaki, Ryuichi Sato, Yuki Haruta, Tadao Nagasaki, Yoshinori Yasui, Yoshinori Hasegawa, Yoshikazu Mutoh, Tomoki Kimura, Tomonori Sato, Reoto Takei, Satoshi Hagimoto, Yoichiro Noguchi, Yasuhiko Yamano, Hajime Sasano, Sho Ota, Yasushi Nakamori, Kazuhisa Yoshiya, Fukuki Saito, Tomoyuki Yoshihara, Daiki Wada, Hiromu Iwamura, Syuji Kanayama, Shuhei Maruyama, Takashi Yoshiyama, Ken Ohta, Hiroyuki Kokuto, Hideo Ogata, Yoshiaki Tanaka, Kenichi Arakawa, Masafumi Shimoda, Takeshi Osawa, Hiroki Tateno, Isano Hase, Shuichi Yoshida, Shoji Suzuki, Miki Kawada, Hirohisa Horinouchi, Fumitake Saito, Keiko Mitamura, Masao Hagihara, Junichi Ochi, Tomoyuki Uchida, Rie Baba, Daisuke Arai, Takayuki Ogura, Hidenori Takahashi, Shigehiro Hagiwara, Genta Nagao, Shunichiro Konishi, Ichiro Nakachi, Koji Murakami, Mitsuhiro Yamada, Hisatoshi Sugiura, Hirohito Sano, Shuichiro Matsumoto, Nozomu Kimura, Yoshinao Ono, Hiroaki Baba, Yusuke Suzuki, Sohei Nakayama, Keita Masuzawa, Shinichi Namba, Ken Suzuki, Yoko Naito, Yu-Chen Liu, Ayako Takuwa, Fuminori Sugihara, James B. Wing, Shuhei Sakakibara, Nobuyuki Hizawa, Takayuki Shiroyama, Satoru Miyawaki, Yusuke Kawamura, Akiyoshi Nakayama, Hirotaka Matsuo, Yuichi Maeda, Takuro Nii, Yoshimi Noda, Takayuki Niitsu, Yuichi Adachi, Takatoshi Enomoto, Saori Amiya, Reina Hara, Yuta Yamaguchi, Teruaki Murakami, Tomoki Kuge, Kinnosuke Matsumoto, Yuji Yamamoto, Makoto Yamamoto, Midori Yoneda, Toshihiro Kishikawa, Shuhei Yamada, Shuhei Kawabata, Noriyuki Kijima, Masatoshi Takagaki, Noah Sasa, Yuya Ueno, Motoyuki Suzuki, Norihiko Takemoto, Hirotaka Eguchi, Takahito Fukusumi, Takao Imai, Munehisa Fukushima, Haruhiko Kishima, Hidenori Inohara, Kazunori Tomono, Kazuto Kato, Meiko Takahashi, Fumihiko Matsuda, Haruhiko Hirata, Yoshito Takeda, Hidefumi Koh, Tadashi Manabe, Yohei Funatsu, Fumimaro Ito, Takahiro Fukui, Keisuke Shinozuka, Sumiko Kohashi, Masatoshi Miyazaki, Tomohisa Shoko, Mitsuaki Kojima, Tomohiro Adachi, Motonao Ishikawa, Kenichiro Takahashi, Takashi Inoue, Toshiyuki Hirano, Keigo Kobayashi, Hatsuyo Takaoka, Kazuyoshi Watanabe, Naoki Miyazawa, Yasuhiro Kimura, Reiko Sado, Hideyasu Sugimoto, Akane Kamiya, Naota Kuwahara, Akiko Fujiwara, Tomohiro Matsunaga, Yoko Sato, Takenori Okada, Yoshihiro Hirai, Hidetoshi Kawashima, Atsuya Narita, Kazuki Niwa, Yoshiyuki Sekikawa, Koichi Nishi, Masaru Nishitsuji, Mayuko Tani, Junya Suzuki, Hiroki Nakatsumi, Takashi Ogura, Hideya Kitamura, Eri Hagiwara, Kota Murohashi, Hiroko Okabayashi, Takao Mochimaru, Shigenari Nukaga, Ryosuke Satomi, Yoshitaka Oyamada, Nobuaki Mori, Tomoya Baba, Yasutaka Fukui, Mitsuru Odate, Shuko Mashimo, Yasushi Makino, Kazuma Yagi, Mizuha Hashiguchi, Junko Kagyo, Tetsuya Shiomi, Satoshi Fuke, Hiroshi Saito, Tomoya Tsuchida, Shigeki Fujitani, Mumon Takita, Daiki Morikawa, Toru Yoshida, Takehiro Izumo, Minoru Inomata, Naoyuki Kuse, Nobuyasu Awano, Mari Tone, Akihiro Ito, Yoshihiko Nakamura, Kota Hoshino, Junichi Maruyama, Hiroyasu Ishikura, Tohru Takata, Toshio Odani, Masaru Amishima, Takeshi Hattori, Yasuo Shichinohe, Takashi Kagaya, Toshiyuki Kita, Kazuhide Ohta, Satoru Sakagami, Kiyoshi Koshida, Kentaro Hayashi, Tetsuo Shimizu, Yutaka Kozu, Hisato Hiranuma, Yasuhiro Gon, Namiki Izumi, Kaoru Nagata, Ken Ueda, Reiko Taki, Satoko Hanada, Kodai Kawamura, Kazuya Ichikado, Kenta Nishiyama, Hiroyuki Muranaka, Kazunori Nakamura, Naozumi Hashimoto, Keiko Wakahara, Sakamoto Koji, Norihito Omote, Akira Ando, Nobuhiro Kodama, Yasunari Kaneyama, Shunsuke Maeda, Takashige Kuraki, Takemasa Matsumoto, Koutaro Yokote, Taka-Aki Nakada, Ryuzo Abe, Taku Oshima, Tadanaga Shimada, Masahiro Harada, Takeshi Takahashi, Hiroshi Ono, Toshihiro Sakurai, Takayuki Shibusawa, Yoshifumi Kimizuka, Akihiko Kawana, Tomoya Sano, Chie Watanabe, Ryohei Suematsu, Hisako Sageshima, Ayumi Yoshifuji, Kazuto Ito, Saeko Takahashi, Kota Ishioka, Morio Nakamura, Makoto Masuda, Aya Wakabayashi, Hiroki Watanabe, Suguru Ueda, Masanori Nishikawa, Yusuke Chihara, Mayumi Takeuchi, Keisuke Onoi, Jun Shinozuka, Atsushi Sueyoshi, Yoji Nagasaki, Masaki Okamoto, Sayoko Ishihara, Masatoshi Shimo, Yoshihisa Tokunaga, Yu Kusaka, Takehiko Ohba, Susumu Isogai, Aki Ogawa, Takuya Inoue, Satoru Fukuyama, Yoshihiro Eriguchi, Akiko Yonekawa, Keiko Kan-o, Koichiro Matsumoto, Kensuke Kanaoka, Shoichi Ihara, Kiyoshi Komuta, Yoshiaki Inoue, Shigeru Chiba, Kunihiro Yamagata, Yuji Hiramatsu, Hirayasu Kai, Koichiro Asano, Tsuyoshi Oguma, Yoko Ito, Satoru Hashimoto, Masaki Yamasaki, Yu Kasamatsu, Yuko Komase, Naoya Hida, Takahiro Tsuburai, Baku Oyama, Minoru Takada, Hidenori Kanda, Yuichiro Kitagawa, Tetsuya Fukuta, Takahito Miyake, Shozo Yoshida, Shinji Ogura, Shinji Abe, Yuta Kono, Yuki Togashi, Hiroyuki Takoi, Ryota Kikuchi, Shinichi Ogawa, Tomouki Ogata, Shoichiro Ishihara, Arihiko Kanehiro, Shinji Ozaki, Yasuko Fuchimoto, Sae Wada, Nobukazu Fujimoto, Kei Nishiyama, Mariko Terashima, Satoru Beppu, Kosuke Yoshida, Osamu Narumoto, Hideaki Nagai, Nobuharu Ooshima, Mitsuru Motegi, Akira Umeda, Kazuya Miyagawa, Hisato Shimada, Mayu Endo, Yoshiyuki Ohira, Masafumi Watanabe, Sumito Inoue, Akira Igarashi, Masamichi Sato, Hironori Sagara, Akihiko Tanaka, Shin Ohta, Tomoyuki Kimura, Yoko Shibata, Yoshinori Tanino, Takefumi Nikaido, Hiroyuki Minemura, Yuki Sato, Yuichiro Yamada, Takuya Hashino, Masato Shinoki, Hajime Iwagoe, Hiroshi Takahashi, Kazuhiko Fujii, Hiroto Kishi, Masayuki Kanai, Tomonori Imamura, Tatsuya Yamashita, Masakiyo Yatomi, Toshitaka Maeno, Shinichi Hayashi, Mai Takahashi, Mizuki Kuramochi, Isamu Kamimaki, Yoshiteru Tominaga, Tomoo Ishii, Mitsuyoshi Utsugi, Akihiro Ono, Toru Tanaka, Takeru Kashiwada, Kazue Fujita, Yoshinobu Saito, Masahiro Seike, Hiroko Watanabe, Hiroto Matsuse, Norio Kodaka, Chihiro Nakano, Takeshi Oshio, Takatomo Hirouchi, Shohei Makino, Moritoki Egi, Yosuke Omae, Yasuhito Nannya, Takafumi Ueno, Kazuhiko Katayama, Masumi Ai, Yoshinori Fukui, Atsushi Kumanogoh, Toshiro Sato, Naoki Hasegawa, Katsushi Tokunaga, Makoto Ishii, Ryuji Koike, Yuko Kitagawa, Akinori Kimura, Seiya Imoto, Satoru Miyano, Seishi Ogawa, Takanori Kanai, Koichi Fukunaga, Yukinori Okada, Akinori Kanai, Yutaka Suzuki, Yoshiya Oda, Yoichiro Kamatani, Ho Namkoong, Ryunosuke Saiki, Akinori Kimura, Ryuji Koike, Seishi Ogawa, Satoru Miyano, Seiya Imoto, Takanori Kanai, Koichi Fukunaga, Yukinori Okada, Anders Mälarstig, Albert Tenesa

**Affiliations:** 1 The Roslin Institute, University of Edinburgh, Easter Bush Campus, Midlothian, UK; 2 Advanced Care Research Centre Academy, University of Edinburgh, Edinburgh, UK; 3 Laboratory of Complex Trait Genomics, Department of Computational Biology and Medical Sciences, Graduate School of Frontier Sciences, The University of Tokyo, Tokyo, Japan; 4 Department of Genome Informatics, Graduate School of Medicine, the University of Tokyo, Tokyo, Japan; 5 Research Fellow, Japan Society for the Promotion of Science, Tokyo, Japan; 6 Department of Medical Epidemiology and Biostatistics, Karolinska Institute, Stockholm, Sweden; 7 Pfizer Research and Development, Stockholm, Sweden; 8 The University of Tokyo, Tokyo, Japan; 9 Institute of Medical Science, The University of Tokyo, Tokyo, Japan; 10 Laboratory of Systems Genomics, Department of Computational Biology and Medical Sciences, Graduate School of Frontier Sciences, The University of Tokyo, Chiba, Japan; 11 Department of Lipidomics, Graduate School of Medicine, The University of Tokyo, Tokyo, Japan; 12 Department of Infectious Diseases, Keio University School of Medicine, Tokyo, Japan; 13 Department of Pathology and Tumor Biology, Kyoto University, Kyoto, Japan; 14 Institute of Science, Tokyo, Japan; 15 Medical Innovation Promotion Center, Tokyo Medical and Dental University, Tokyo, Japan; 16 Institute for the Advanced Study of Human Biology (WPI-ASHBi), Kyoto University, Kyoto, Japan; 17 Department of Medicine, Center for Hematology and Regenerative Medicine, Karolinska Institute, Stockholm, Sweden; 18 M&D Data Science Center, Institute of Science, IIR, Tokyo, Japan; 19 Division of Health Medical Intelligence, Human Genome Center, the Institute of Medical Science, The University of Tokyo, Tokyo, Japan; 20 Division of Gastroenterology and Hepatology, Department of Medicine, Keio University School of Medicine, Tokyo, Japan; 21 AMED-CREST, Japan Agency for Medical Research and Development, Tokyo, Japan; 22 Division of Pulmonary Medicine, Department of Medicine, Keio University School of Medicine, Tokyo, Japan; 23 Department of Statistical Genetics, Osaka University Graduate School of Medicine, Suita, Japan; 24 Laboratory of Statistical Immunology, Immunology Frontier Research Center (WPI-IFReC), Osaka University, Suita, Japan; 25 Laboratory for Systems Genetics, RIKEN Center for Integrative Medical Sciences, Yokohama, Japan; 26 MRC Human Genetics Unit at the MRC, Institute of Genetics and Molecular Medicine, University of Edinburgh, Western General Hospital, Edinburgh, UK; 27 Department of Infectious Diseases, Keio University School of Medicine, Tokyo, Japan; 28 Department of Respiratory Medicine and Clinical Immunology, Osaka University Graduate School of Medicine, Suita, Japan; 29 Laboratory of Veterinary Infectious Disease, School of Veterinary Medicine, Kitasato University, Aomori, Japan; 30 Genomics Unit, Keio Cancer Center, Keio University Hospital, Tokyo, Japan; 31 Integrated Frontier Research for Medical Science Division, Institute for Open and Transdisciplinary Research Initiatives, Osaka University, Suita, Japan; 32 Division of Gastroenterology and Hepatology, Department of Medicine, Keio University School of Medicine, Tokyo, Japan; 33 M&D Data Science Center, Tokyo Medical and Dental University, Tokyo, Japan; 34 Department of Pathology, Graduate School of Medicine, Yokohama City University, Yokohama, Japan; 35 Human Immunology, Single Cell Genomics, WPI Immunology Frontier Research Center, Osaka University, Suita, Japan; 36 Genome Information Research Center, Research Institute for Microbial Diseases, Osaka University, Suita, Japan; 37 Department of Biomedical Informatics, Harvard Medical School, Boston, MA USA; 38 Division of Pathology, Yokohama Municipal Citizen’s Hospital, Yokohama, Japan; 39 Division of Infectious Disease, Yokohama Municipal Citizen’s Hospital, Yokohama, Japan; 40 Division of Health Medical Intelligence, Human Genome Center, the Institute of Medical Science, the University of Tokyo, Tokyo, Japan; 41 Department of Immunopathology, Immunology Frontier Research Center (WPI-IFReC), Osaka University, Suita, Japan; 42 Department of Respiratory Medicine, Juntendo University Faculty of Medicine and Graduate School of Medicine, Tokyo, Japan; 43 Department of General Medicine, Juntendo University Faculty of Medicine and Graduate School of Medicine, Tokyo, Japan; 44 Department of Emergency and Disaster Medicine, Juntendo University Faculty of Medicine and Graduate School of Medicine, Tokyo, Japan; 45 Department of Internal Medicine and Rheumatology, Juntendo University Faculty of Medicine and Graduate School of Medicine, Tokyo, Japan; 46 Department of Nephrology, Juntendo University Faculty of Medicine and Graduate School of Medicine, Tokyo, Japan; 47 Atopy (Allergy) Research Center, Juntendo University Graduate School of Medicine, Tokyo, Japan; 48 Department of Emergency and Critical Care Medicine, Keio University School of Medicine, Tokyo, Japan; 49 Department of Anesthesiology, Keio University School of Medicine, Tokyo, Japan; 50 Department of Laboratory Medicine, Keio University School of Medicine, Tokyo, Japan; 51 Keio University Health Center, Tokyo, Japan; 52 Department of Respiratory Medicine, Saitama Cardiovascular and Respiratory Center, Kumagaya, Japan; 53 JCHO (Japan Community Health Care Organization) Saitama Medical Center, Internal Medicine, Saitama, Japan; 54 Department of Respiratory Medicine, Tokyo Women’s Medical University, Tokyo, Japan; 55 Department of General Medicine, Tokyo Women’s Medical University, Tokyo, Japan; 56 Clinical Research Center, Tokyo Medical and Dental University Hospital of Medicine, Tokyo, Japan; 57 Department of Medical Informatics, Tokyo Medical and Dental University Hospital of Medicine, Tokyo, Japan; 58 Respiratory Medicine, Tokyo Medical and Dental University, Tokyo, Japan; 59 Clinical Laboratory, Tokyo Medical and Dental University Hospital of Medicine, Tokyo, Japan; 60 Department of Internal Medicine, Kawasaki Municipal Ida Hospital, Kawasaki, Japan; 61 Department of Respiratory Medicine, Osaka Saiseikai Nakatsu Hospital, Osaka, Japan; 62 Department of Infection Control, Osaka Saiseikai Nakatsu Hospital, Osaka, Japan; 63 Department of Infectious Diseases, Tosei General Hospital, Seto, Japan; 64 Department of Respiratory, Allergic Diseases Internal Medicine, Tosei General Hospital, Seto, Japan; 65 Department of Emergency and Critical Care Medicine, Kansai Medical University General Medical Center, Moriguchi, Japan; 66 Fukujuji Hospital, Kiyose, Japan; 67 Department of Pulmonary Medicine, Saitama City Hospital, Saitama, Japan; 68 Department of Infectious Diseases, Saitama City Hospital, Saitama, Japan; 69 Department of General Thoracic Surgery, Saitama City Hospital, Saitama, Japan; 70 Department of Pulmonary Medicine, Eiju General Hospital, Tokyo, Japan; 71 Division of Infection Control, Eiju General Hospital, Tokyo, Japan; 72 Department of Hematology, Eiju General Hospital, Tokyo, Japan; 73 Saiseikai Utsunomiya Hospital, Utsunomiya, Japan; 74 Department of Respiratory Medicine, Tohoku University Graduate School of Medicine, Sendai, Japan; 75 Department of Infectious Diseases, Tohoku University Graduate School of Medicine, Sendai, Japan; 76 Department of Respiratory Medicine, Kitasato University Kitasato Institute Hospital, Tokyo, Japan; 77 Core Instrumentation Facility, Immunology Frontier Research Center and Research Institute for Microbial Diseases, Osaka University, Suita, Japan; 78 Laboratory of Human Immunology (Single Cell Immunology), Immunology Frontier Research Center, Osaka University, Suita, Japan; 79 Laboratory of Immune Regulation, Immunology Frontier Research Center, Osaka University, Suita, Japan; 80 Department of Pulmonary Medicine, Faculty of Medicine, University of Tsukuba, Tsukuba, Japan; 81 Department of Neurosurgery, Faculty of Medicine, the University of Tokyo, Tokyo, Japan; 82 Department of Integrative Physiology and Bio-Nano Medicine, National Defense Medical College, Tokorozawa, Japan; 83 Department of Head and Neck Surgery, Aichi Cancer Center Hospital, Nagoya, Japan; 84 Department of Otorhinolaryngology-Head and Neck Surgery, Osaka University Graduate School of Medicine, Suita, Japan; 85 Department of Neurosurgery, Osaka University Graduate School of Medicine, Suita, Japan; 86 Department of Otolaryngology and Head and Neck Surgery, Kansai Rosai Hospital, Hyogo, Japan; 87 Division of Infection Control and Prevention, Osaka University Hospital, Suita, Japan; 88 Department of Biomedical Ethics and Public Policy, Osaka University Graduate School of Medicine, Suita, Japan; 89 Center for Genomic Medicine, Kyoto University Graduate School of Medicine, Kyoto, Japan; 90 Tachikawa Hospital, Tachikawa, Japan; 91 Department of Emergency and Critical Care Medicine, Tokyo Women’s Medical University Medical Center East, Tokyo, Japan; 92 Department of Medicine, Tokyo Women’s Medical University Medical Center East, Tokyo, Japan; 93 Department of Pediatrics, Tokyo Women’s Medical University Medical Center East, Tokyo, Japan; 94 Internal Medicine, Sano Kosei General Hospital, Sano, Japan; 95 Japan Community Health Care Organization Kanazawa Hospital, Kanazawa, Japan; 96 Department of Respiratory Medicine, Saiseikai Yokohamashi Nanbu Hospital, Yokohama, Japan; 97 Department of Clinical Laboratory, Saiseikai Yokohamashi Nanbu Hospital, Yokohama, Japan; 98 Department of Respiratory Medicine, Japan Organization of Occupational Health and Safety, Kanto Rosai Hospital, Kawasaki, Japan; 99 Department of General Internal Medicine, Japan Organization of Occupational Health and Safety, Kanto Rosai Hospital, Kawasaki, Japan; 100 Ishikawa Prefectural Central Hospital, Kanazawa, Japan; 101 Kanagawa Cardiovascular and Respiratory Center, Yokohama, Japan; 102 Department of Respiratory Medicine, National Hospital Organization Tokyo Medical Center, Tokyo, Japan; 103 Department of Allergy, National Hospital Organization Tokyo Medical Center, Tokyo, Japan; 104 Department of General Internal Medicine and Infectious Diseases, National Hospital Organization Tokyo Medical Center, Tokyo, Japan; 105 Department of Respiratory Medicine, Toyohashi Municipal Hospital, Toyohashi, Japan; 106 Keiyu Hospital, Yokohama, Japan; 107 Department of Respiratory Medicine, KKR Sapporo Medical Center, Sapporo, Japan; 108 Division of General Internal Medicine, Department of Internal Medicine, St Marianna University School of Medicine, Kawasaki, Japan; 109 Department of Emergency and Critical Care Medicine, StMarianna University School of Medicine, Kawasaki, Japan; 110 Japanese Red Cross Medical Center, Tokyo, Japan; 111 Matsumoto City Hospital, Matsumoto, Japan; 112 Department of Emergency and Critical Care Medicine, Faculty of Medicine, Fukuoka University, Fukuoka, Japan; 113 Department of Infection Control, Fukuoka University Hospital, Fukuoka, Japan; 114 Department of Rheumatology, National Hospital Organization Hokkaido Medical Center, Sapporo, Japan; 115 Department of Respiratory Medicine, National Hospital Organization Hokkaido Medical Center, Sapporo, Japan; 116 Department of Emergency and Critical Care Medicine, National Hospital Organization Hokkaido Medical Center, Sapporo, Japan; 117 NHO Kanazawa Medical Center, Kanazawa, Japan; 118 Division of Respiratory Medicine, Department of Internal Medicine, Nihon University School of Medicine, Tokyo, Japan; 119 Musashino Red Cross Hospital, Musashino, Japan; 120 Division of Respiratory Medicine, Social Welfare Organization Saiseikai Imperial Gift Foundation, Inc, Saiseikai Kumamoto Hospital, Kumamoto, Japan; 121 Department of Respiratory Medicine, Nagoya University Graduate School of Medicine, Nagoya, Japan; 122 Department of Internal Medicine, Fukuoka Tokushukai Hospital, Kasuga, Japan; 123 Department of Endocrinology, Hematology and Gerontology, Chiba University Graduate School of Medicine, Chiba, Japan; 124 Department of Emergency and Critical Care Medicine, Chiba University Graduate School of Medicine, Chiba, Japan; 125 National Hospital Organization Kumamoto Medical Center, Kumamoto, Japan; 126 Division of Infectious Diseases and Respiratory Medicine, Department of Internal Medicine, National Defense Medical College, Tokorozawa, Japan; 127 Sapporo City General Hospital, Sapporo, Japan; 128 Department of Internal Medicine, Tokyo Saiseikai Central Hospital, Tokyo, Japan; 129 Department of Pulmonary Medicine, Tokyo Saiseikai Central Hospital, Tokyo, Japan; 130 Department of Respiratory Medicine, Fujisawa City Hospital, Fujisawa, Japan; 131 Uji-Tokushukai Medical Center, Uji, Japan; 132 Department of Infectious Disease and Clinical Research Institute, National Hospital Organization Kyushu Medical Center, Fukuoka, Japan; 133 Department of Respirology, National Hospital Organization Kyushu Medical Center, Fukuoka, Japan; 134 Division of Respirology, Rheumatology, and Neurology, Department of Internal Medicine, Kurume University School of Medicine, Kurume, Japan; 135 Department of Infectious Disease, National Hospital Organization Kyushu Medical Center, Fukuoka, Japan; 136 Ome Municipal General Hospital, Ome, Japan; 137 Graduate School of Medical Sciences, Research Institute for Diseases of the Chest, Kyushu University, Fukuoka, Japan; 138 Department of Medicine and Biosystemic Science, Kyushu University Graduate School of Medical Sciences, Fukuoka, Japan; 139 Daini Osaka Police Hospital, Osaka, Japan; 140 Department of Emergency and Critical Care Medicine, Faculty of Medicine, University of Tsukuba, Tsukuba, Japan; 141 Department of Hematology, Faculty of Medicine, University of Tsukuba, Tsukuba, Japan; 142 Department of Nephrology, Faculty of Medicine, University of Tsukuba, Tsukuba, Japan; 143 Department of Cardiovascular Surgery, Faculty of Medicine, University of Tsukuba, Tsukuba, Japan; 144 Division of Pulmonary Medicine, Department of Medicine, Tokai University School of Medicine, Isehara, Japan; 145 Department of Anesthesiology and Intensive Care Medicine, Kyoto Prefectural University of Medicine, Kyoto, Japan; 146 Department of Infection Control and Laboratory Medicine, Kyoto Prefectural University of Medicine, Kyoto, Japan; 147 Department of Respiratory Internal Medicine, St Marianna University School of Medicine, Yokohama-City Seibu Hospital, Yokohama, Japan; 148 KINSHUKAI Hanwa The Second Hospital, Osaka, Japan; 149 Gifu University School of Medicine Graduate School of Medicine, Emergency and Disaster Medicine, Gifu, Japan; 150 Department of Respiratory Medicine, Tokyo Medical University Hospital, Tokyo, Japan; 151 JA Toride Medical Hospital, Toride, Japan; 152 Okayama Rosai Hospital, Okayama, Japan; 153 Himeji St Mary’s Hospital, Himeji, Japan; 154 Emergency & Critical Care, Niigata University, Niigata, Japan; 155 Emergency & Critical Care Center, National Hospital Organization Kyoto Medical Center, Kyoto, Japan; 156 National Hospital Organization Tokyo Hospital Hospital, Kiyose, Japan; 157 Fujioka General Hospital, Fujioka, Japan; 158 Department of General Medicine, School of Medicine, International University of Health and Welfare Shioya Hospital, Ohtawara, Japan; 159 Department of Pharmacology, School of Pharmacy, International University of Health and Welfare Shioya Hospital, Ohtawara, Japan; 160 Department of Respiratory Medicine, International University of Health and Welfare Shioya Hospital, Ohtawara, Japan; 161 Department of Clinical Laboratory, International University of Health and Welfare Shioya Hospital, Ohtawara, Japan; 162 Department of Cardiology, Pulmonology, and Nephrology, Yamagata University Faculty of Medicine, Yamagata, Japan; 163 Division of Respiratory Medicine and Allergology, Department of Medicine, School of Medicine, Showa University, Tokyo, Japan; 164 Department of Pulmonary Medicine, Fukushima Medical University, Fukushima, Japan; 165 Kansai Electric Power Hospital, Osaka, Japan; 166 Division of Infectious Diseases, Kumamoto City Hospital, Kumamoto, Japan; 167 Department of Respiratory Medicine, Kumamoto City Hospital, Kumamoto, Japan; 168 Department of Emergency and Critical Care Medicine, Tokyo Metropolitan Police Hospital, Tokyo, Japan; 169 Department of Respiratory Medicine, Gunma University Graduate School of Medicine, Maebashi, Japan; 170 National Hospital Organization Saitama Hospital, Wako, Japan; 171 Tokyo Medical University Ibaraki Medical Center, Inashiki, Japan; 172 Department of Internal Medicine, Kiryu Kosei General Hospital, Kiryu, Japan; 173 Department of Pulmonary Medicine and Oncology, Graduate School of Medicine, Nippon Medical School, Tokyo, Japan; 174 Division of Respiratory Medicine, Tsukuba Kinen General Hospital, Tsukuba, Japan; 175 Division of Respiratory Medicine, Department of Internal Medicine, Toho University Ohashi Medical Center, Tokyo, Japan; 176 Division of Anesthesiology, Department of Surgery Related, Kobe University Graduate School of Medicine, Kobe, Japan; 177 Genome Medical Science Project (Toyama), National Center for Global Health and Medicine, Tokyo, Japan; 178 Department of Biomolecular Engineering, Graduate School of Tokyo Institute of Technology, Tokyo, Japan; 179 Laboratory of Viral Infection, Department of Infection Control and Immunology, Ōmura Satoshi Memorial Institute & Graduate School of Infection Control Sciences, Kitasato University, Tokyo, Japan; 180 Department of Insured Medical Care Management, Tokyo Medical and Dental University Hospital of Medicine, Tokyo, Japan; 181 Division of Immunogenetics, Department of Immunobiology and Neuroscience, Medical Institute of Bioregulation, Kyushu University, Fukuoka, Japan; 182 Center for Infectious Disease Education and Research (CiDER), Osaka University, Suita, Japan; 183 Department of Organoid Medicine, Keio University School of Medicine, Tokyo, Japan; 184 Department of Surgery, Keio University School of Medicine, Tokyo, Japan; 185 Institute of Research, Tokyo Medical and Dental University, Tokyo, Japan

**Keywords:** Proteome profiles, Sex-dimorphic protein quantitative trait loci, Sex differences, Heritable quantitative trait, Quantitative trait

## Abstract

Males and females exhibit differences in proteome profiles associated with disease risk. However, sex-dimorphic protein quantitative trait loci (SD-pQTL) and their effects on sex differences in health disorders have not been thoroughly investigated. We conducted a sex-stratified, genome-wide association study on 2,922 proteins using data from 30,272 individuals of Caucasian ancestry from the UK Biobank and compared the estimated effects on protein levels of these variants in the men and women to identify SD-pQTLs. The identified SD-pQTLs were replicated using data from two Japanese cohorts (comprising 2,886 and 1,394 individuals, respectively), as well as from 1,990 Finnish, 630 South Asian, and 662 Black ancestry individuals. Sex-dimorphic pleiotropy and the causal relationship between protein levels and health disorders were assessed using the identified SD-pQTLs. We identified 113 SD-pQTLs associated with 65 proteins. Of the 113 SD-pQTLs, 52 were significant in both sexes, five were not significant in either sex, and 42 and 14 were significant only in males and females, respectively. Variant rs2270416 was significantly associated with the CDH15 protein in both sexes but showed opposite effect direction in men and women. Of the 113 SD-pQTLs identified, a total of 41 were replicated in a meta-analysis encompassing Japanese, South Asian, and Black ancestry individuals. SD-pQTLs for proteins APOE (rs157581) and SNAP25 (rs4420638) exhibited sex-dimorphic associations with dementia, indicating sex dimorphic pleiotropy in both proteins and health disorders. From sex-stratified Mendelian randomization using the SD-pQTLs, proteins NCAM1 and PZP showed significant causal relationship with dementia in males and females, respectively. The present study provides evidence of sex-dimorphic genetic architecture in protein-level regulation, elucidating the proteo-genetic architecture for sex differences in human variation.

**Supplementary Information:**

The online version contains supplementary material available at 10.1038/s41598-025-10031-z.

## Introduction

Accounting for sex differences at the molecular level could lead to better personalized disease prediction, diagnosis, and treatment, as well as an improved understanding of the biological mechanisms driving these differences. Mounting evidence suggests differences between males and females in various aspects of health disorders, including the effects of risk factors, prevalence, and disease outcomes^1–13^. At the molecular level, sex-differentiated architectures and effects have been investigated at the genomic, transcriptomic, and epigenomic levels, providing insight into sex differences^14–25^.

In recent years, there has been growing evidence of sex differences in protein levels and the differential effects of genetic variants on protein levels^26–28^. For example, a study involving 1,277 European brains indicated differences in protein expression between sexes, as well as genetic variants affecting protein levels differently according to sex^28^. Another study involving 800 individuals from a Dutch population reported sex-dimorphic genetic regulation of inflammatory proteins, providing broad insights into sex differences in proteo-genetic architecture^29^. These studies have, however, been limited in terms of the number of traits and proteins studied as well as the number of individuals and ancestries represented.

The objectives of the present study were to identify sex-dimorphic protein quantitative trait loci (SD-pQTLs) and examine their association with sex differences in health disorders. To achieve this, we analyzed 2,922 proteins using data from 30,272 individuals of Caucasian ancestry from the UK Biobank. Next, we replicated the identified SD-pQTLs using five different datasets: (1) 2,886 and (2) 1,394 individuals of Japanese ancestry from the BioBank Japan and the Japan COVID-19 Task Force, respectively, (3) 1,990 individuals of Finnish ancestry from FinnGen, as well as (4) 630 individuals of South Asian ancestry (Indian, Pakistani, and Bangladeshi) and (5) 662 individuals of Black ancestry (African and Caribbean) from the UK Biobank. In addition, we assessed the sex-dimorphic effects of SD-pQTLs on health disorders by conducting sex-stratified GWAS for 30 long-term conditions using 338,568 individuals, distinct from those included in the proteomics analysis.

## Results

### Sex differences in proteome profiles and regulation

A total of 30,272 individuals were included in the proteome analysis. The mean age ± SD was 57.1 ± 7.9 years. 46.2% (13,974 individuals; mean age ± SD: 57.3 ± 8.1) were males and 53.8% (16,298; 57.0 ± 7.8) were females. Of the 2,922 proteins, 2,249 showed a significant association with sex (false discovery rate (FDR)-corrected p-value, termed q, < 0.05, Supplementary Table 1). To estimate the heritability of proteins in each sex and the genetic correlation of proteins between the sexes, we assessed the effect of variants on blood protein levels within each sex by performing sex-stratified genome-wide association study (GWAS) using the 30,272 individuals. As a result, 1,612 proteins showed significant heritability in both males and females. 194 and 348 proteins showed sex-specific significant heritability in males and females, respectively (q < 0.05, Supplementary Table 1). 1,818 proteins showed a significant genetic correlation differing from zero between males and females (q < 0.05, Supplementary Table 1).

### Identification of sex-dimorphic pQTL

After selecting index variants from the sex-stratified GWAS using linkage disequilibrium clumping, 31,753 and 36,979 pQTLs were identified in males and females, respectively (q < 0.05, Supplementary Tables 2 and 3). By comparing the effects of variants between males and females across the genome, we derived 113 index pQTLs that exhibit significantly different effects on the levels of 65 proteins by sex, henceforth termed sex-dimorphic pQTLs, or SD-pQTLs (q < 0.05, Supplementary Table 4). 25 out of the 113 SD-pQTLs were not significant in the sex-combined GWAS, which was conducted on protein levels without sex stratification. 52 out of the 113 SD-pQTLs were significant in both sexes, while 42 and 14 were significant only in males and females, respectively (q < 0.05). The remaining five SD-pQTLs were sex-dimorphic but not significant in either sex. Among the 52 SD-pQTLs exhibiting a significant effect in both sexes, variant rs2270416, associated with CDH15, was a stop-gain variant in CDH15 and the only variant with a sex-antagonistic effect. rs2270416 was masked in the sex-agnostic analysis, as it was not significant in the sex-combined GWAS (sex-dimorphic test: q = 2.07E-27; beta in males: -0.23, q = 4.63E-11; beta in females: 0.26, q = 3.01E-17; sex-combined test q = 0.957).

### Replication of the identified SD-pQTL

**Fig. 1:**
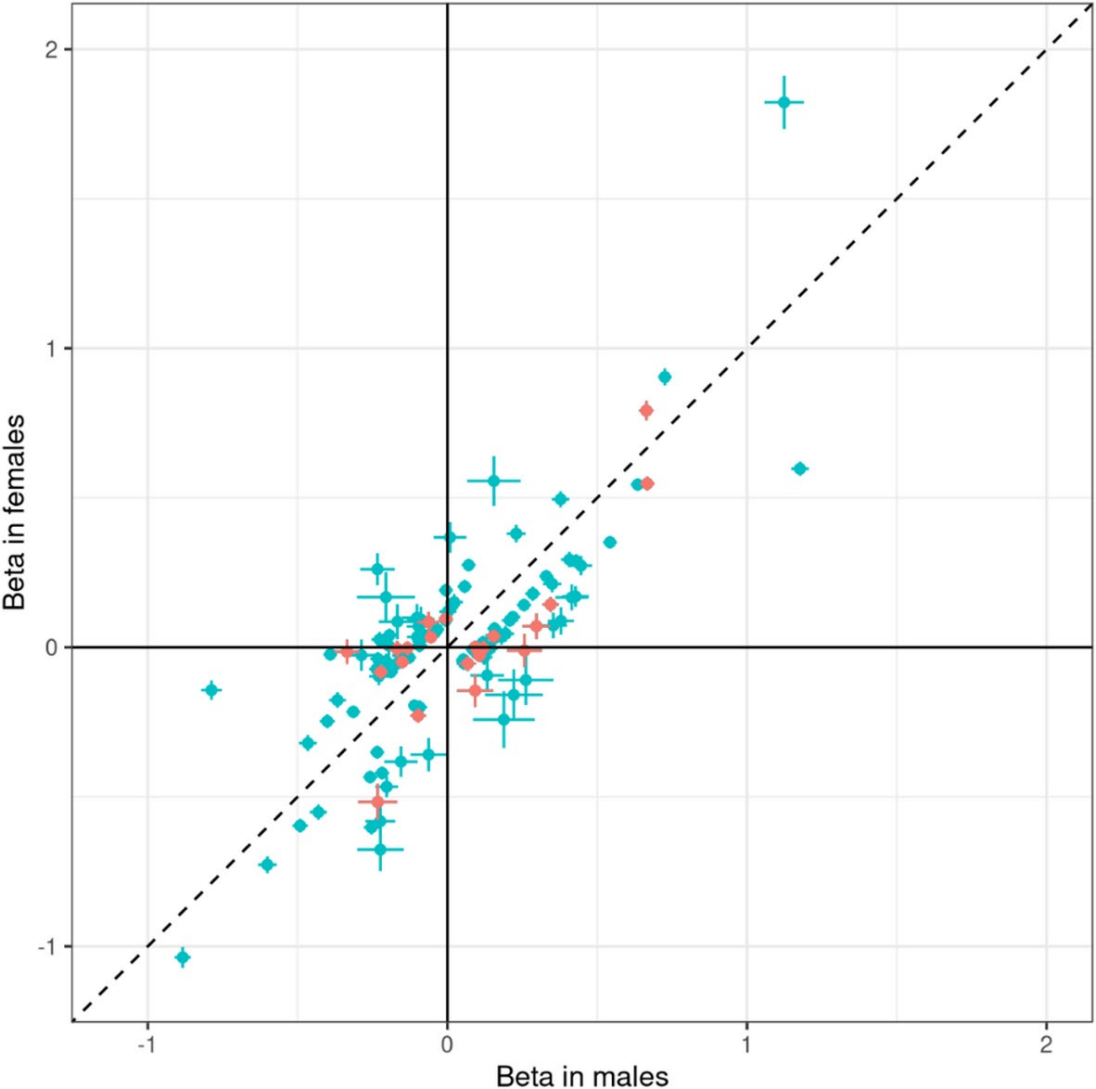
The effects of variants on protein levels in males and females. Each point represents a variant, while lines indicating the 95% confidence interval. The beta values and confidence intervals correspond to the UK Biobank Caucasian ancestry analysis results. Points are highlighted in red when the sex-dimorphic p-value is more significant in the multi-ancestry meta-analysis than in the Caucasian-only UK-Biobank analysis.

To assess the confidence of the identified SD-pQTLs, we conducted sex-stratified analyses using independent datasets of multiple ancestries. In the BioBank Japan dataset, only 76 out of the 113 SD-pQTLs could be tested because either the proteins were not available or the genetic variants were not polymorphic in BioBank Japan. Among the 76 SD-pQTLs, 12 exhibited significant sex-dimorphic (q-value < 0.05) effects on the protein levels. In the Japan COVID-19 Task Force dataset, 80 out of the 113 SD-pQTLs were testable. Among the 80 SD-pQTLs, three showed significant sex-dimorphic effects. In the FinnGen dataset, 13 of the 111 tested SD-pQTLs showed significant effects. In the UK Biobank South Asian samples, two out of 90 tested SD-pQTLs were significant. In the UK Biobank Black samples, none of the 113 tested SD-pQTLs were significant (Supplementary Table 4). One SD-pQTL (Protein PAEP: variant rs67944) was replicated in BioBank Japan, Japan COVID-19 Task Force, FinnGen, and UK Biobank South Asian samples. Two SD-pQTLs (EDDM3B: rs12890226 and LEFTY2: rs360076) were replicated in BioBank Japan and Japan COVID-19 Task Force. Seven SD-pQTLs (INSL3: rs1044303, KLK3: rs10993994, rs2569747, rs266849, rs266869, PLA2G2A: rs12044628, and PLB1: rs34590437) were replicated only in BioBank Japan, nine (DDR1: rs1264344, EDDM3B: rs4982354, KLK4: rs79486581, NCAM1: rs748631, PAEP: rs10858128, PZP: rs11048434, rs2277413, SUSD4: rs7526539, and TEX101: rs7259375, rs35033974, rs2355990) were replicated only in FinnGen, and one (PZP: rs11615443) was replicated only in UK Biobank South Asian samples. A meta-analysis encompassing all datasets resulted in 22 out of the 113 SD-pQTLs yielding higher confidence than the UK Biobank analysis alone, suggesting that these 22 SD-pQTLs are robust associations (Fig. 1). The sex-dimorphic effect of the variant rs2270416 on CDH15 did not show higher significance in the meta-analysis (p-value = 4.18E-06) than in the UK Biobank alone (p-value = 2.89E-35).

### SD-pQTLs’ sex-dimorphic effect on health disorders

**Fig. 2:**
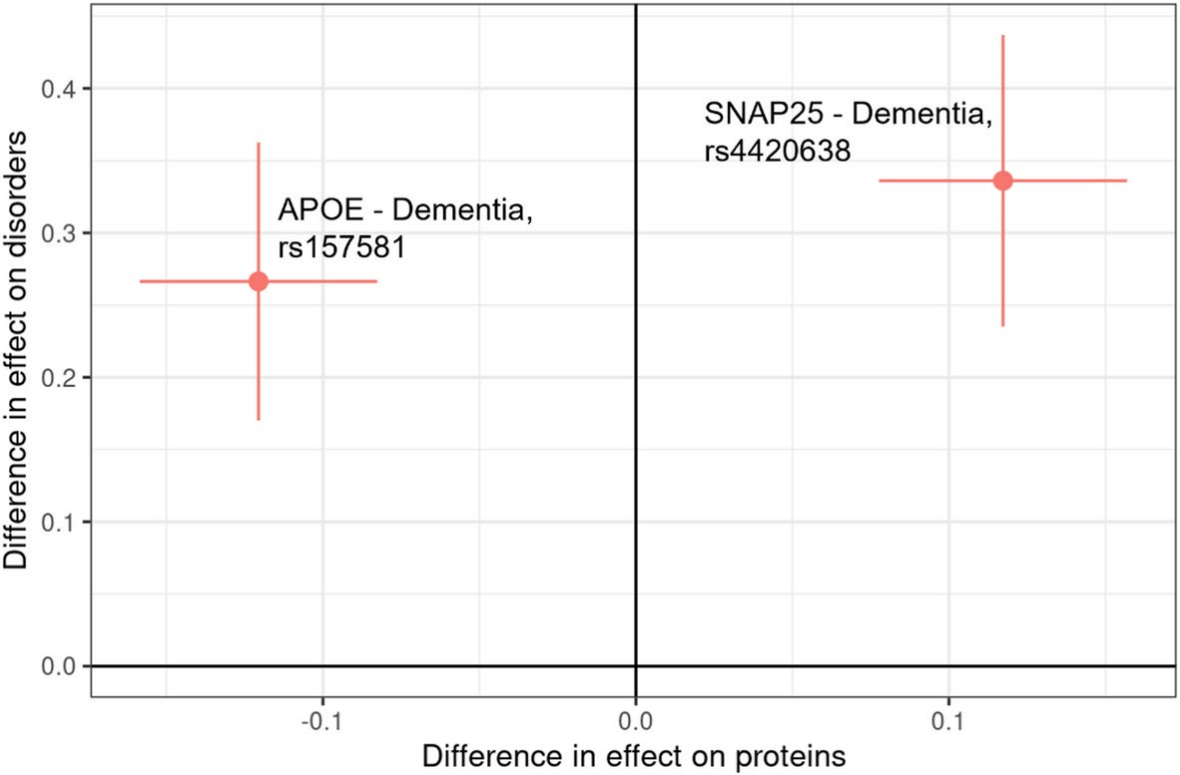
Sex dimorphic pleiotropy between proteins and health disorders. Scatterplot of variants’ sex-dimorphic effect on proteins and health disorders. Each point represents a variant, with lines indicating the 95% confidence interval. The X-axis and Y-axis represents the difference in the effect on protein and health disorders, between males and females, respectively, with a higher value indicating a higher effect in females. The label on each point describes the associated protein and health disorder for that variant.

Genetic variants exhibiting a sex-dimorphic effect on protein levels might also influence health disorders in a sex-dimorphic manner, highlighting the importance of considering sex differences in genetic studies for improving precision medicine and understanding disease mechanisms. To investigate whether the 113 SD-pQTLs in this study also exhibit sex-dimorphic effects on health disorders, we first conducted sex-stratified GWAS on 30 long-term conditions using the 338,568 Caucasian individuals, independent of the samples used in the proteomic GWAS analyses (Supplementary Tables 5 and 6). We then derived results for the 113 SD-pQTLs. As a result, two out of the 113 SD-pQTLs exhibited a significant sex-dimorphic effect on health disorders (p-value < 1.47E-05, Bonferroni corrected p-value accounting for the 113 SD-pQTL and the 30 long-term conditions). SD-pQTLs of the proteins APOE (rs157581, chr19: 45,395,714) and SNAP25 (rs4420638, chr19: 45,422,946) exhibited a sex dimorphic effect on dementia, suggesting potential sex-dimorphic pleiotropy involving these proteins and dementia (Fig. 2). Although it did not reach a significant threshold after correction for multiple testing, the variant rs2270416, which showed a sex dimorphic effect on CDH15, also exhibited a suggested sex dimorphic effect on depression (p-value = 3.29E-02). Sex-specific survival analysis between APOE and SNAP25 measured plasma protein levels and prospective dementia showed a significant association for APOE both in males (hazard ratio (HR) = 0.68, p-value = 3.89E-10) and females (HR = 0.67, p-value = 5.64E-13), but with non-significant sex-dimorphic effect (p-value = 0.94). SNAP25 showed a significant association with prospective dementia only in females (HR = 1.24, p-value = 3.17E-04) but not in males (HR = 1.01, p-value = 0.838).

**Fig. 3:**
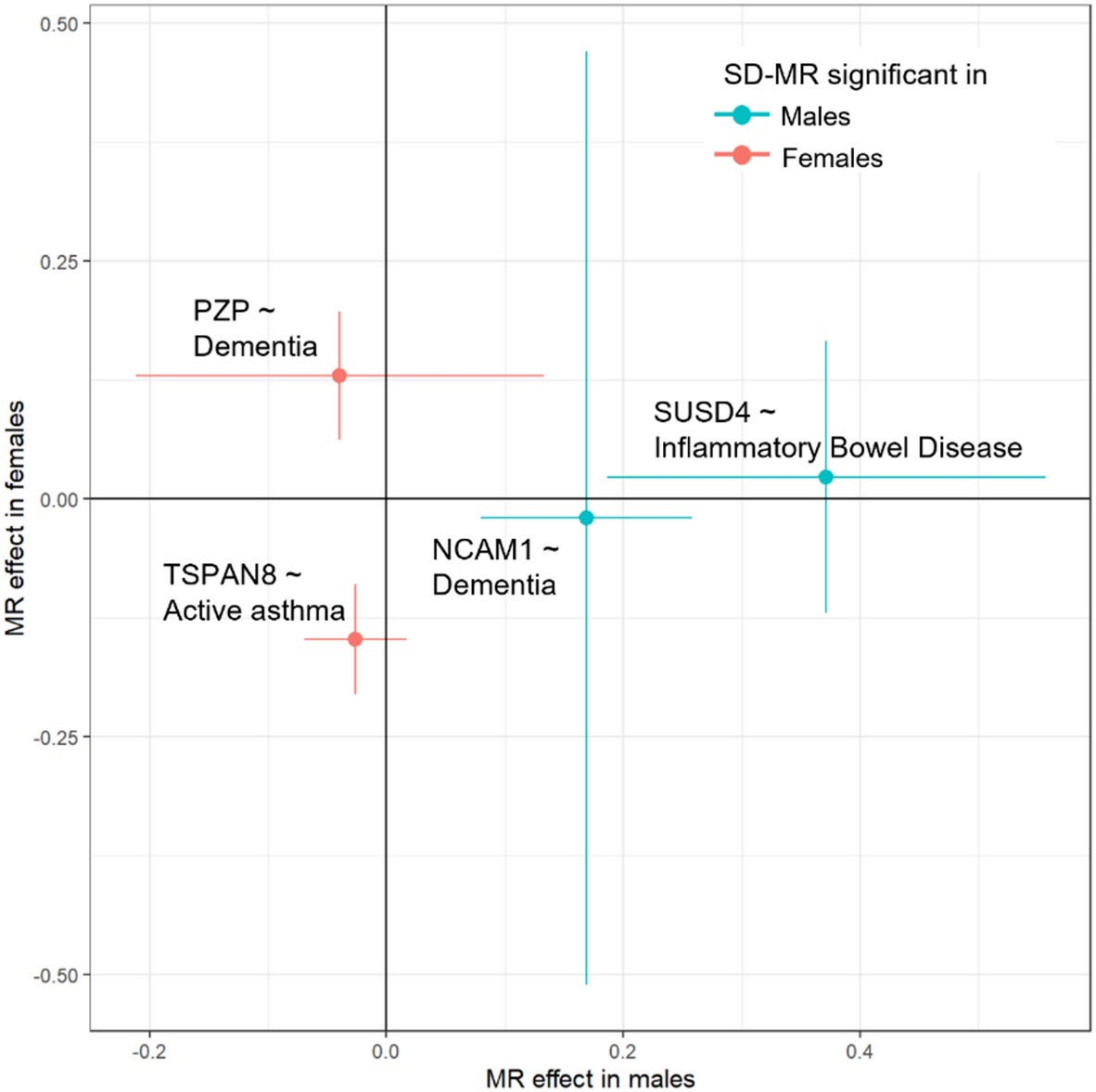
Sex specific protein-disorder causal relationships from SD-pQTLs. Scatterplot of sex-stratified MR for protein-disorder pairs. Each point represents a pair, with lines indicating the 95% confidence interval. The X-axis and Y-axis represent causal estimates in males and females, respectively. Blue-coloured pairs indicate male-specific relationships, while red coloured pairs indicate female-specific relationships. The label on each point provides the protein and health disorder of the pair.

We also questioned whether proteins can, in a causal way, differentially influence disease risk depending on sex. This can shed light on possible sex-dimorphic disease mechanisms, that are potentially responsible for sex disparities in drug effectiveness and safety. One such example is the higher risk of adverse drug reactions observed in females, which has been attributed to sex-agnostic drug prescription practices^30^. Mendelian randomisation (MR) can infer causal relationships between an exposure, such as protein level, and a disease outcome using genetic variants as instrumental variables. That is, genetic variants that are associated with the outcome only through their effect on the exposure. Rather than mere correlation, where no cause-and-effect relationship is implied, MR can estimate causality between exposure and outcome. This is achieved by leveraging the random allocation of genetic variants at birth, thus minimising confounding and reverse causation from outcome to exposure. In light of this, we investigated possible sex-dimorphic causal effects of proteins on health disorders by conducting sex-stratified MR analyses using the identified SD-pQTLs. Here, we conducted MR using seven proteins that have more than one valid SD-pQTL in both sexes and 30 long-term conditions (Fig. 3 and Supplementary Table 7). In contrast to our previous analysis of sex-dimorphic effects on disorders (Fig. 2), sex-stratified MR has the advantages to assess causality and whether or not SD-pQTL effects on disorders are driven by pleiotropy. We identified four protein-disorder pairs where a causal relationship was observed in only one sex. For male-specific relationships, SUSD4-inflammatory bowel disease (Inverse variance weighted (IVW) fixed effects meta-analysis MR estimate q-value in males = 0.038, in females = 1.00) and NCAM1-dementia (q-value in males = 0.045, q-value in females = 1.00) pairs were identified. For female-specific relationships, TSPAN8-asthma (beta in females = 0.84, q-value in females = 2.52E-04) and PZP-dementia (beta in females = 0.96, q-value in females = 0.040) pairs were identified. We did not observe protein-disorder pairs where the male and female-specific causal estimates differed significantly (t-test between male and female estimates q-value > 0.05). No protein-disorder pair showed evidence of heterogeneity or pleiotropy (Supplementary Tables 8 and 9). For the four protein-disorder pairs identified through sex-specific MR, we further fit sex-specific survival analyses. We did not find a significant association between protein levels and prospective disease onset of the tested health disorders in either males or females. Cox Proportional Hazard models were fit for SUSD4-inflammatory bowel disease (protein HR p-value in males = 0.52, in females = 0.35), NCAM1-dementia (HR p-value in males = 0.76, HR p-value in females = 0.61), TSPAN8-asthma (HR p-value in males = 0.37, HR p-value in females = 0.66), PZP-dementia (HR p-value in males = 0.87, HR p-value in females = 0.41). In summary, we identified protein-disorder pairs causally affecting health disorder risk in a male-only or female-only way. No protein-disorder pairs showed significant difference in their sex-stratified causal effects here. These findings may guide future research on potential sex-specific role of those proteins in disease pathogenesis.

## Discussion

Our study investigated sex differences in the proteo-genetic architecture of blood proteins across ancestries, providing a comprehensive assessment at a depth and breadth not previously achieved. The findings provide insight into different relationships between genetic variants, protein levels, and health disorders depending on sex. The replication of the identified SD-pQTLs across independent datasets of various ancestries strengthens the validity of our findings.

Our analysis revealed SD-pQTLs that exhibited various differential effects on protein levels between males and females, such as sex-specific or sex-antagonistic effects, as reported in previous sex-stratified GWAS conducted on various human phenotypes^16^. This suggests that sex differences at the molecular level have complex background constituted of various mechanism that cannot be explained solely by sex-specific or sex-antagonistic pathways. One notable finding from this study is variant rs2270416, which is significantly associated in both sexes, yet exhibits opposite effect directions with CDH15 plasma protein level. This is the only such case in this study, although sex-specific or dimorphic effect of CDH15 have not been clearly suggested to date. It may be implied that this antagonistic association is specific to the individuals in the UK Biobank, considering the lower significance in the meta-analysis or a potential false positive. Hence, further investigation would be required on the SD-pQTLs identified in this study to assess whether the sex-dimorphic effect of each variant is shared or differs across ancestries.

We also found that some SD-pQTLs have been identified as significant pQTLs from sex-agnostic GWAS. This implies that effect size estimates from sex-agnostic GWAS may be inaccurate, as they can represent an average between significantly different effects in males and females. Furthermore, significant associations in sex-agnostics GWAS may reflect strong effects in only one sex, which would go undetected without sex-stratified analysis. These findings highlight the importance of performing sex-stratified GWAS in uncovering sex-dimorphic genetic effects that may be missed in sex-agnostic studies.

In addition, our study provides insights into the sex-dimorphic effects of SD-pQTLs on diseases risk. For instance, we identified SD-pQTLs associated with dementia, exhibiting sex dimorphic effect on disease risk. Blood levels of APOE and SNAP25 have been reported to be associated with dementia and suggested as biomarkers for dementia^31–34^. Further investigation with the identified SD-pQTL of APOE and SNAP25 in this study could provide insight into the pathophysiology of dementia and help improve its prediction, diagnosis, and treatment. Additionally, our Mendelian randomization analysis using SD-pQTLs revealed sex-specific causal relationships between proteins and health disorders. Specifically, the NCAM1 and PZP proteins showed sex-specific causal relationships with dementia in males and females, respectively. This finding may provide insight into sex-specific pathophysiology of dementia through further investigation of their biological pathways. Blood levels of these two proteins have previously been reported to be associated with dementia^35–37^. Here, we provide additional genetically informed support for these associations.

PZP is a protein initially described as a major pregnancy-associated protein, showing elevated levels during pregnancy and higher expression levels in females than in males^38,39^. It has been reported that only females showed elevated serum PZP levels prior to the onset of Alzheimer’s disease, in line with the female-specific causal relationship between PZP and dementia observed in the present study^37^. Plasma NCAM1 has also been found to mediate sex-related neurodegeneration differences in cognitively normal adults, potentially suggesting that NCAM1 exerts sex-different neuropathological effects at a pre-dementia stage as well^40^. A previous study of temporal lobe proteomes reported a significant upregulation of NCAM1 only in the brains of male dementia patients^41^. Although protein levels can vary between brain and blood tissues, the male-specific association between brain NCAM1 levels and dementia reported in the previous study of temporal lobe proteomes and the male-specific causal relationship between serum NCAM1 and dementia in the present study could provide illuminating insight into the sex-dimorphic pathophysiology of dementia. Additionally, NCAM1 has been implicated in the mechanism of action of antidepressants, particularly in response to duloxetine treatment for depression^42,43^. Studies of pooled data from seven randomised clinical trials found no significant sex differences in duloxetine’s efficacy, safety or tolerability in treating major depressive disorder^44,45^. However, these duloxetine studies did not evaluate response based on drug dosage or plasma concentrations, which could mask dose-dependent sex differences. Further study on a large cohort, accompanied by experimental analysis, would be necessary to dissect the detailed role of these proteins in dementia. Taken together, these findings highlight the potential role of sex-dimorphic genetic regulation in disease pathogenesis and its implication for sex-specific precision medicine in prediction, diagnosis, and treatment.

However, the results of this study should be interpreted with caution, as we utilized only a limited number of blood proteins currently available, which is just a subset of human proteome. The SD-pQTLs and their associations with health disorders also require careful consideration, as the analysis was limited to Caucasian individuals from the UK Biobank, leveraging its large sample size. Additionally, disease prevalence differs between males and females, as observed in our dataset. Furthermore, the MR investigation of sex-dimorphic causal relationship should also be interpreted with caution, as this study relied solely on SD-pQTLs. Further studies using additional valid genetic instruments for each disease are needed to better dissect sex-dimorphic effects in causal inference. Another limitation of our MR analysis is that it was based on individuals of Caucasian ancestry from the UK Biobank, as SD-pQTLs from other ancestries would have limited power in an MR framework. Restricting MR analyses to ancestrally homogenous samples reduces the risk of population stratification that can lead to violation of the independence and exclusion restriction MR assumptions^46^. However, transferring MR results across ancestries is challenging due to differences in LD patterns and allele frequencies^46^, although methods to facilitate trans-ancestry MR have been proposed^47^. It should also be noted that aside from population stratification, MR analyses can be biased by assortative mating, dynastic events (the direct effect of one’s parents on a phenotype), or selection bias, such as participation bias in the UK Biobank. Such processes can confound the relationship between SD-pQTLs and disease outcomes, potentially violating the MR independence assumption^48^. Finally, out MR analysis does not account for potential time-varying effects of proteins on disease risk, which may be important when considering the timing of intervention^46^. Additionally, the limited statistical support in the survival analysis maybe due to a small sample size, specifically the low number of incident dementia events following the date the blood sample was collected for the protein level measurement (288 males and 302 females). Notably, the survival analyses of PZP and NCAM1 on dementia showed the same direction of effect as in the MR, suggesting the possibility of insufficient sample size. Therefore, larger studies with longer follow-up are needed to clarify the sex-dimorphic associations between protein levels and disease incidence observed in this study.

## Conclusions

Our study provides comprehensive findings, resources, and insights into sex differences in the proteo-genetic architecture of proteins and their relationships with disease susceptibility. Further research is needed to investigate the underlying mechanisms of sex-dimorphic protein regulation, their links to diseases, and their potential for therapeutic applications.

## Methods

### Data sources

The UK Biobank is a prospective research resource of population-based cohort study that include comprehensive phenotype and genotype data from approximately 500,000 participants recruited in 2006–2010 residing in England, Scotland, and Wales (www.ukbiobank.ac.uk). This open-access resource was established to support investigations into the factors influencing various health outcomes^49^. We utilized genotyped data and recently released proteome data from the UK Biobank^26^. To ensure homogeneity, we limited analyses to unrelated individuals of Caucasian genetic ancestry (UK Biobank Data-Field 22006) with less than 10% missing genotypes and those with matched recorded sex (Data-Field 31) and genetically determined sex (Data-Field 22001). We used this matched sex information to define sex in subsequent analyses. The unrelated participants were identified and extracted using the KING software with following options: --unrelated –degree 2 (version 2.28)^50^. Autosomal and X-chromosomal genotypes of the selected individuals were filtered using PLINK software (version 1.90b) with the following options; --geno 0.01, --hwe 1e-15, --maf 0.01, and mind 0.1, retaining 539,158 variants^51^. These filtered variants were used in the subsequent analyses. To ensure homogeneity in proteome analysis, we extracted proteome data of randomly selected baseline participants from protein batches 0–6, which are highly representative of the UK Biobank overall^26^. Following these selections, the final dataset for proteome analysis consisted of 13,974 males and 16,298 females, totalling 30,272 individuals. The remaining individuals without proteome data, 156,581 males and 181,987 females, totalling 338,568 individuals, were retained, and utilized in subsequent sex-stratified analyses on health disorders.

The BioBank Japan (https://biobankjp.org/en/) project (first cohort) is a hospital-based cohort study that recruited approximately 200,000 patients with at least one of the 47 complex diseases between 2003 and 2007 across 66 hospitals in Japan^52^. Proteomic profiling was performed on unrelated individuals of East-Asian ancestry from two previous studies with whole genome sequencing datasets, using the Olink Explore 3072 panel following the manufacturer’s protocol^53,54^. Proteomic data processing and data quality control were conducted according to Olink protocols. The rank-based inverse normal transformation was applied to protein level measurements before association tests for males and females, respectively. Sex-stratified pQTL summary statistics of serum protein levels in the BioBank Japan project were obtained by meta-analysing results for each sex derived from each study separately using REGENIE v3.2.9 (adjusted for age, age2, proteomic profiling batch, and the first 10 genetic principal components) and METAL (fixed-effect inverse variance weighted)^55,56^. In total, 2,886 participants (including 2,151 males and 735 females) were included in the sex-stratified pQTL analyses in BioBank Japan.

The Japan COVID-19 Task Force (JCTF) was established in early 2020 as a nationwide multicentre consortium to overcome the COVID-19 pandemic (https://www.covid19-taskforce.jp/en/home/). Plasma protein expression was measured using the Olink Explore 3072 platform. We bridge-normalized the Normalized Protein eXpression (NPX) values using the OlinkAnalyze R package with 16 intersecting samples as bridging samples. Samples with QC warning flags were removed. Genotyping was performed using Infinium Asian Screening Array (Illumina, CA, USA), and stringent sample and variant level quality control (QC) filters were applied (sample QC: sample call rate < 0.98, samples of estimated non East Asian ancestry based on PCA with HapMap project samples, variant QC: variant call rate < 0.99, minor allele count < 5, p-value for Hardy-Weinberg equilibrium < 1e-10, and with more than 5% allele frequency difference when compared with the representative reference panels of Japanese ancestry^57^. We performed genome-wide genotype imputation, by using SHAPEIT4 software version 4.2.1 for haplotype phasing and Minimac4 software version 1.0.1 for genotype imputation^58,59^. For imputation, we used our in-house and Japanese-specific reference panel composed of *N* = 4,561 whole-genome sequence (WGS) data from multiple studies (e.g., *N* = 1,939 from the BBJ study and *N* = 141 WGS from the previous study)^60,61^. The final dataset consisted of 995 males and 399 females, totalling 1,394 individuals. The genomic coordinates of the variants are based on the genome build GRCh37 throughout this study.

FinnGen (https://www.finngen.fi/en) is a public-private research project, combining genome and digital healthcare data on ~ 500,000 Finns that launched in 2017. The nation-wide research project is a pre-competitive partnership of Finnish biobanks and their background organisations (universities and university hospitals) and international pharmaceutical industry partners and Finnish biobank cooperative (FINBB). FinnGen aims to provide novel medically and therapeutically relevant insight into human diseases and is described in detail in their flagship paper^62^. FinnGen partners are listed in full here: https://www.finngen.fi/en/partners.

### Investigating sex different genetics in blood proteome

To investigate the effect of sex on proteins, multivariable regression was conducted accounting for age, age^2^, body mass index, UK Biobank Centre, UK Biobank genetic array, time between blood sampling and measurement, and the first 20 genetic principal components. To investigate genetic effects on blood proteins by sex, we conducted sex-stratified GWAS on protein levels using the proteome data from the 30,272 selected Caucasian individuals and compared variants’ effect on protein between males and females. For the sex-stratified GWAS, REGENIE software (version 3.2.2) was utilized with sex-stratified protein level normalization using --apply-rint option^55^. Covariates considered in the GWAS included age, age^2^, batch, UK Biobank Centre, UK Biobank genetic array, time between blood sampling and measurement, and the first 20 genetic principal components. Sex, Sex*age, and sex*age^2^ were added in covariates for sex-combined GWAS. Protein GLIPR1 was excluded from the analysis due to exceptionally high percent of failing QC (99.40%) during the protein measurement^26^.

We estimated variant-based heritability as the variance explained by the genetic variants’ effect using the LD-score regression^63^. To estimate sex difference in heritability, we applied t-statistics utilized in a previous sex stratified study^16^. We estimated genetic correlation of each protein between males and females using the High-Definition Likelihood method^64^. To account for multiple testing, we adjusted the p-values using the Benjamini-Hochberg method for each analysis^65^.

### Identification and replication of sex-dimorphic pQTL

To identify variants with different effects between males and females, we compared the effects of each variant across the genome for each protein using two-tailed Student’s t-test, applied in previous sex-stratified GWAS comparison studies^16,66,67^. To select index variants in each significant locus, we utilized PLINK software with following options: --clump, --clump-p1 0.00000005, --clump-p2 0.001, --clump-r2 0.2, --clump-kb 10,000, considering sex-different p-value as the variant’s significance. Clumping was also applied to GWAS results from each sex as well^51^. To account for multiple testing, we adjusted the sex different p-values using the Benjamini-Hochberg method, and index variants with false discovery rate < 0.05 were considered significant and presented as the identified SD-pQTLs in this study^65^. The functional consequences of variants were annotated using Variant Effect Predictor and Ensembl GRCh37 release 112^68^.

To ensure the confidence of the identified SD-pQTLs, we replicated the identified SD-pQTLs in each independent dataset comprised of 2,886 individuals (2,151 males and 735 females) of Japanese ancestry from the BioBank Japan, 1,394 individuals (995 males and 399 females) of Japanese ancestry from the Japan COVID-19 Task Force, 1,990 (958 males and 1,032 females) of Finnish ancestry from FinnGen, as well as 630 individuals (336 males and 294 females) of South Asian (Indian, Pakistani, and Bangladeshi) and 662 individuals (290 males and 372 females) of Black ancestries (Caribbean and African) in the UK Biobank based on the self-reported ancestry (Data-Field 21000), using the protocols from the identification step in this study. For FinnGen, the 1,990 individuals had plasma proteomics measured using the Olink Explore 3072 panel. Quality control of the proteomics data was carried out in accordance with Olink recommendations by the core FinnGen analysis team. GWAS of plasma protein levels were carried out separately by sex using REGENIE [Ref]. NPX values were rank-based inverse normal transformed (--apply-rint) within sex. Age, batch, genotyping array and genetic PCs (1–5) were included as covariates. A detailed description of FinnGen genotype data, its quality control and pre-processing has been described previously^62^. FinnGen data freeze 12 was used for this analysis and sex-specific pQTL results were downloaded from the FinnGen sandbox under approved proposal F_2024_056. The replicated results were combined using a random-effect meta-analysis, conducted with the metafor R package and set to “REML”, to obtain a single estimate^69^. Variants were considered replicated SD-pQTLs if the p-value from the meta-analysis was more significant than that from the UK Biobank analysis alone, indicating a consistent trend in sex-different effect of the variant across independent datasets of multiple ancestries.

### Sex dimorphic effect of SD-pQTL on health disorders

To investigate SD-pQTLs’ sex dimorphic effect on health disorder, we firstly conducted sex-stratified GWAS on 30 predefined long-term conditions (Supplementary Table 5) using the 338,568 individuals retained during sample selection^70^. We then derived results for the 113 SD-pQTLs. For the sex-stratified GWAS, we used the REGENIE software with the following covariates: age, age^2^, UK Biobank Centre, UK Biobank genetic array, and the first 20 genetic principal components^55^. Sex-different effects on health disorders were derived using the method applied in SD-pQTL analysis in this study. SD-pQTLs were considered to have sex-different pleiotropy with health disorders if the SD-pQTL exhibited p-value for sex dimorphic effect on health disorder lower than 1.47E-05, which 0.05 divided by the number of long-term conditions and the number of SD-pQTLs, accounting for multiple tests.

To investigate sex-dimorphic causal relationships between plasma protein levels and the 30 predefined health disorders, we employed a two-sample mendelian randomisation (MR) approach separately for males and females^71^. MR analyses are based on the use of genetic variants as instrumental variables (IV). IVs are variables associated with an exposure but not with the outcome of interest through any other pathway. Three assumptions are required for MR to be valid: (1) IVs are significantly associated with the exposure (the relevance assumption); (2) there are no confounders of the IVs and the outcome (the independence assumption); and (3) IVs do not affect the outcome other than through the exposure (the exclusion restriction assumption)^71^.

We used the SD-pQTLs identified in this study as instrumental variables. For each MR analysis between protein and health disorder by sex, SD-pQTLs that were significantly associated with each protein (FDR < 0.05) and were not significantly associated with each health disorder (p-value ≥ 5E-8) were selected. SD-pQTLs were used regardless of their genomic position relative to their associated protein’s gene location, i.e. both cis- and trans-pQTLs. Only proteins with more than one SD-pQTLs after filtering were considered for the MR analysis. This was done in order to allow the use of sensitivity analyses testing for horizontal pleiotropy and heterogeneity^46^.

MR was conducted using the TwoSampleMR R package (version 0.5.11)^72^. The causal estimates were initially derived using the inverse-variance weighed (IVW) fixed effects meta-analysis method, accompanied by weighted median (WM) and MR-Egger methods^73–75^. WM and MR-Egger analyses were conducted to the proteins with more than 2 valid SD-pQTLs. Sensitivity analyses to test potential horizontal pleiotropy of the causal relationships and heterogeneity of the instrumental variables were conducted using the MR-Egger intercept test and the Cochran’s Q statistic test, respectively^75–77^. Sex dimorphic causal relationships were identified using a two-tailed Student’s t-test and considered as sex-dimorphic if FDR for the sex dimorphic effect is below 0.05. Causal relationships with FDR < 0.05 for either the pleiotropy or heterogeneity tests were excluded.

For the protein-disorder pairs that were significant in the sex-stratified MR analysis, we further examined whether UKB protein levels could predict incident health disorder survival in a sex-dimorphic way. For this purpose, we employed Cox Proportional Hazards models separately for each sex, using baseline protein levels as predictors and time to first incident diagnosis of the respective health disorder as outcome. Survival times were derived based on up to 16 years of follow-up health records. Models were adjusted for age at protein measurement, as it is a confounding factor for many age-related health disorders. Individuals with a prevalent health disorder diagnosis before, or up to 1 year after protein measurement were excluded. The “survival” R package (version 3.7-0) was used to fit the Cox Proportional Hazards models^78^. All methods have been carried out in accordance with relevant guidelines and regulations.

## Electronic supplementary material

Below is the link to the electronic supplementary material.

Supplementary Material 1

Supplementary Material 2

## Data Availability

Data of the UK Biobank project in our study is publicly available upon request and approval. Details of procedures for accessing the UK Biobank data can be found here: https://www.ukbiobank.ac.uk/enable-your-research/apply-for-access. The GWAS summary statistics from the UK Biobank dataset and analysis scripts used in this study are publicly available from the University of Edinburgh repository: https://doi.org/10.7488/ds/7917. Whole genome sequence data of BioBank Japan project in this study is available at NBDC database (https://humandbs.dbcls.jp/hum0014-v33). Proteome data and clinical information is available upon request and approval. Details of procedures for accessing Biobank Japan data can be found here: https://biobankjp.org/en/researchers/1974#gsc.tab=0. The summary statistics of pQTL in JCTF will be available upon publication.

## Abbreviations

pQTL: Protein quantitative trait loci
SD-pQTL: Sex-dimorphic protein quantitative trait loci
GWAS: Genome-wide association study
MR: Mendelian Randomization
APOE: apolipoprotein E
NCAM1: Neural cell adhesion molecule 1
PZP: Pregnancy Zone Protein
SNAP25: Synaptosome Associated Protein 25
