# Supplementary material for: Identification and replication of sex-dimorphic protein quantitative trait loci across multiple ancestries and their associations with diseases
Source: Sci Rep. 2025 Aug 28;15:31721. doi: 10.1038/s41598-025-10031-z (PMC12394655; doi:10.1038/s41598-025-10031-z)
Supplement: Supplementary file 1 — Supplementary Material 1 [file 41598_2025_10031_MOESM1_ESM.pdf]

STROBE-MR checklist of recommended items to address in reports of Mendelian randomization studies[1 2]

Item No.	Section		Checklist item	Page No.	Relevant text from manuscript
1	**TITLE and ABSTRACT**		Indicate Mendelian randomization (MR) as the study's design in the title and/or the abstract if that is a main purpose of the study	3	Sex-dimorphic pleiotropy and causal relationship between protein levels and health disorders were assessed using the identified SD-pQTLs.
	**INTRODUCTION**				
2	**Background**		Explain the scientific background and rationale for the reported study. What is the exposure? Is a potential causal relationship between exposure and outcome plausible? Justify why MR is a helpful method to address the study question	10, Supplementary Table 5	We also questioned whether proteins can, in a causal way, differentially influence disease risk depending on sex.
3	**Objectives**		State specific objectives clearly, including pre-specified causal hypotheses (if any). State that MR is a method that, under specific assumptions, intends to estimate causal effects	10	Page 10: We also questioned whether proteins can, in a causal way, differentially influence disease risk depending on sex.Mendelian Randomisation (MR) can infer causal relationships between an exposure, such as protein level, and a disease outcome using genetic variants as instrumental variables.
	**METHODS**				
4	**Study design and data sources**		Present key elements of the study design early in the article. Consider including a table listing sources of data for all phases of the study. For each data source contributing to the analysis, describe the following:		
		a)	Setting: Describe the study design and the underlying population, if possible. Describe the setting, locations, and relevant dates, including periods of recruitment, exposure, follow-up, and data collection, when available.	15	The UK Biobank is a prospective research resource of population-based cohort study that include comprehensive phenotype and genotype data from approximately 500,000 participants recruited in 2006–2010 residing in England, Scotland, and Wales (www.ukbiobank.ac.uk). This open-access resource was established to support investigations into the factors influencing various health outcomes
		b)	Participants: Give the eligibility criteria, and the sources and methods of selection of participants. Report the sample size, and whether any power or sample size calculations were carried out prior to the main analysis	16	Following these selections, the final dataset for proteome analysis consisted of 13,974 males and 16,298 females, totalling 30,272 individuals. The remaining individuals without proteome data, 156,581 males and 181,987 females, totalling 338,568 individuals, were retained, and

				utilized in subsequent sex-stratified analyses on health disorders.
	c)	Describe measurement, quality control and selection of genetic variants	16,18,20	Page 16: Autosomal and X-chromosomal genotypes of the selected individuals were filtered using PLINK software (version 1.90b) with the following options; --geno 0.01, --hwe 1e-15, --maf 0.01, and mind 0.1, retaining 539,158 variants […]<br><br>Page 18: To identify variants with different effects between males and females, we compared the effects of each variant across genome for each protein using two-tailed Student’s t-test, applied in previous sex-stratified GWAS comparison studies [16, 62, 63]. To select index variants in each significant locus, we utilized PLINK software with following options: --clump, --clump-p1 0.00000005, --clump-p2 0.001, --clump-r2 0.2, --clump-kb 10000, considering sex-different p-value as the variant’s significance. Clumping was also applied to GWAS results from each sex as well [48]. To account for multiple testing, we adjusted the sex different p-values using the Benjamini-Hochberg method, and index variants with false discovery rate < 0.05 were considered significant and presented as the identified SD-pQTLs in this study [61]. […]<br><br>Page 20: For each MR analysis between protein and health disorder by sex, SD-pQTLs that were significantly associated with each protein (FDR < 0.05) and were not significantly associated with each health disorder (p-value ≥ 5E-8) were selected. Only proteins with more than one SD-pQTLs after filtering were considered for the MR analysis.
	d)	For each exposure, outcome, and other relevant variables, describe methods of assessment and diagnostic criteria for diseases	15, 19, Supplementary Table 5	Page 15: We utilized genotyped data and recently released proteome data from the UK Biobank [26].<br><br>Page 19: To investigate SD-pQTLs’ sex dimorphic effect on health disorder, we firstly conducted sex-stratified GWAS on 30 predefined long-term conditions (Supplementary table 5) […]<br><br>.

		e)	Provide details of ethics committee approval and participant informed consent, if relevant	21	The UK Biobank project was approved by the National Research Ethics Service Committee North West-Haydock (REC reference: 11/NW/0382).
5	**Assumptions**		Explicitly state the three core IV assumptions for the main analysis (relevance, independence and exclusion restriction) as well assumptions for any additional or sensitivity analysis	21	MR analyses are based on the use of genetic variants as instrumental variables (IV). IVs are variables associated with an exposure but not with the outcome of interest through any other pathway[91,92]. Three assumptions are required for MR to be valid: (1) IVs are significantly associated with the exposure (the relevance assumption); (2) there are no confounders of the IVs and the outcome (the independence assumption); and (3) IVs do not affect the outcome other than through the exposure (the exclusion restriction assumption) [66].
6	**Statistical methods: main analysis**		Describe statistical methods and statistics used		
		a)	Describe how quantitative variables were handled in the analyses (i.e., scale, units, model)	16	Proteomic data processing and data quality control were conducted according to Olink protocols. The rank-based inverse normal transformation was applied to protein level measurements before association tests for males and females, respectively
		b)	Describe how genetic variants were handled in the analyses and, if applicable, how their weights were selected	20	For each MR analysis between protein and health disorder by sex, SD-pQTLs that were significantly associated with each protein (FDR < 0.05) and were not significantly associated with each health disorder (p-value ≥ 5E-8) were selected. Only proteins with more than one SD-pQTLs after filtering were considered for the MR analysis. This was done in order to keep proteins with increased genetic variance explained by the exposure, and to allow the use of sensitivity analyses testing for horizontal pleiotropy and heterogeneity
		c)	Describe the MR estimator (e.g. two-stage least squares, Wald ratio) and related statistics. Detail the included covariates and, in case of two-sample MR, whether the same covariate set was used for adjustment in the two samples	17,18,21	Page 17: Covariates considered in the GWAS included age, age2, batch, UK BioBank Centre, UK BioBank genetic array, time between blood

				sampling and measurement, and the first 20 genetic principal components.<br>Page 18: For the sex-stratified GWAS, we used the REGENIE software with the following covariates: age, age2, UK BioBank Centre, UK BioBank genetic array, and the first 20 genetic principal components<br>Page 21: The causal estimates were initially derived using the inverse-variance weighed (IVW) fixed effects meta-analysis method, accompanied by weighted median (WM) and MR-Egger methods [68-70].
		d) Explain how missing data were addressed	15, 18	Page 15: To ensure homogeneity, we limited analyses to unrelated individuals of European White ancestry with less than 10% missing genotypes […] genotypes of the selected individuals were filtered using PLINK software (version 1.90b) with the following options; --geno 0.01, --hwe 1e-15, --maf 0.01, and mind 0.1, retaining 539,158 variants [48].<br>Page 20: Only proteins with more than one SD-pQTLs after filtering were considered for the MR analysis.
		e) If applicable, indicate how multiple testing was addressed	18	Sex dimorphic causal relationships were identified using a two-tailed Student’s t-test and considered as sex-dimorphic if FDR for the sex dimorphic effect is below 0.05. Causal relationships with FDR < 0.05 for either the pleiotropy or heterogeneity tests were excluded.
7	**Assessment of assumptions**	Describe any methods or prior knowledge used to assess the assumptions or justify their validity	NA	NA
8	**Sensitivity analyses and additional analyses**	Describe any sensitivity analyses or additional analyses performed (e.g. comparison of effect estimates from different approaches, independent replication, bias analytic techniques, validation of instruments, simulations)	20	Sensitivity analyses to test potential horizontal pleiotropy of the causal relationships and heterogeneity of the instrumental variables were conducted using the MR-Egger intercept test and the Cochran's Q statistic test, respectively
9	**Software and pre-registration**			

		a) Name statistical software and package(s), including version and settings used	20	MR was conducted using the TwoSampleMR R package (version 0.5.11)
		b) State whether the study protocol and details were pre-registered (as well as when and where)	NA	NA
	**RESULTS**			
10	**Descriptive data**			
		a) Report the numbers of individuals at each stage of included studies and reasons for exclusion. Consider use of a flow diagram	15	We utilized genotyped data and recently released proteome data from the UK Biobank[26]To ensure homogeneity, we limited analyses to unrelated individuals of European White ancestry with less than 10% missing genotypes and those with matched recorded sex and genetically determined sex. The unrelated participants were identified and extracted using the KING software with following options: --unrelated –degree 2 (version 2.28) [47]. Autosomal and X-chromosomal genotypes of the selected individuals were filtered using PLINK software (version 1.90b) with the following options; --geno 0.01, --hwe 1e-15, --maf 0.01, and mind 0.1, retaining 539,158 variants [48]. To ensure homogeneity in proteome analysis, we extracted proteome data of randomly selected baseline participants from protein batches 0-6, which are highly representative of the UK Biobank overall [26]. Following these selections, the final dataset for proteome analysis consisted of 13,974 males and 16,298 females, totalling 30,272 individuals. The remaining individuals without proteome data, 156,581 males and 181,987 females, totalling 338,568 individuals, were retained, and utilized in subsequent sex-stratified analyses on health disorders.
		b) Report summary statistics for phenotypic exposure(s), outcome(s), and other relevant variables (e.g. means, SDs, proportions)	Supplementary tables 1 and 5	
		c) If the data sources include meta-analyses of previous studies, provide the assessments of heterogeneity across these studies	NA	NA
		d) For two-sample MR:<br>i. Provide justification of the similarity of the genetic variant-exposure associations between the exposure and outcome samples	15-16	To ensure homogeneity, we limited analyses to unrelated individuals of European White ancestry with less than 10% missing genotypes and those with matched recorded sex and

			ii. Provide information on the number of individuals who overlap between the exposure and outcome studies		genetically determined sex. The unrelated participants were identified and extracted using the KING software with following options: --unrelated –degree 2 (version 2.28) [47]. Autosomal and X-chromosomal genotypes of the selected individuals were filtered using PLINK software (version 1.90b) with the following options; --geno 0.01, --hwe 1e-15, --maf 0.01, and mind 0.1, retaining 539,158 variants [48]. To ensure homogeneity in proteome analysis, we extracted proteome data of randomly selected baseline participants from protein batches 0-6, which are highly representative of the UK Biobank overall [26]. Following these selections, the final dataset for proteome analysis consisted of 13,974 males and 16,298 females, totalling 30,272 individuals. The remaining individuals without proteome data, 156,581 males and 181,987 females, totalling 338,568 individuals, were retained, and utilized in subsequent sex-stratified analyses on health disorders.
11	**Main results**				
		a)	Report the associations between genetic variant and exposure, and between genetic variant and outcome, preferably on an interpretable scale	Supplementary tables 2, 3, and 6	
		b)	Report MR estimates of the relationship between exposure and outcome, and the measures of uncertainty from the MR analysis, on an interpretable scale, such as odds ratio or relative risk per SD difference	Supplementary table 7	
		c)	If relevant, consider translating estimates of relative risk into absolute risk for a meaningful time period	NA	NA
		d)	Consider plots to visualize results (e.g. forest plot, scatterplot of associations between genetic variants and outcome versus between genetic variants and exposure)	NA	NA
12	**Assessment of assumptions**				
		a)	Report the assessment of the validity of the assumptions	Supplementary table 9	
		b)	Report any additional statistics (e.g., assessments of heterogeneity across genetic variants, such as I^2, Q statistic or E-value)	Supplementary table 8	

13	**Sensitivity analyses and additional analyses**				
		a)	Report any sensitivity analyses to assess the robustness of the main results to violations of the assumptions	Supplementary table 8 and 9	
		b)	Report results from other sensitivity analyses or additional analyses	Supplementary table 8 and 9	
		c)	Report any assessment of direction of causal relationship (e.g., bidirectional MR)	NA	
		d)	When relevant, report and compare with estimates from non-MR analyses	NA	NA
		e)	Consider additional plots to visualize results (e.g., leave-one-out analyses)	NA	NA
	**DISCUSSION**				
14	**Key results**		Summarize key results with reference to study objectives	10	We identified four protein-disorder pairs where a causal relationship was observed in only one of the sexes. For male-specific relationships, SUSD4-inflammatroy bowel disease (Inverse variance weighted (IVW) fixed effects meta-analysis MR estimate q-value in males = 0.038, in females = 1.00) and NCAM1-dementia (q in males = 0.045, q in females = 1.00) pairs were identified. For female-specific relationships, TSPAN8-asthma (beta in females = 0.84, q in females = 2.52E-04) and PZP-dementia (beta in females = 0.96, q in females = 0.040) pairs were identified. We did not observe protein-disorder pairs where the male and female-specific causal estimates differed significantly (t-test between male and female estimates q-value > 0.05).
15	**Limitations**		Discuss limitations of the study, taking into account the validity of the IV assumptions, other sources of potential bias, and imprecision. Discuss both direction and magnitude of any potential bias and any efforts to address them	14	Furthermore, the MR investigation of sex-dimorphic causal relationship should also be interpreted with caution, as this study relied solely on SD-pQTLs. Further studies using additional valid genetic instruments for each disease are needed to better dissect sex-dimorphic effects in causal inference
16	**Interpretation**				

		a) Meaning: Give a cautious overall interpretation of results in the context of their limitations and in comparison with other studies	14	Furthermore, the MR investigation of sex-dimorphic causal relationship should also be interpreted with caution, as this study relied solely on SD-pQTLs. Further studies using additional valid genetic instruments for each disease are needed to better dissect sex-dimorphic effects in causal
		b) Mechanism: Discuss underlying biological mechanisms that could drive a potential causal relationship between the investigated exposure and the outcome, and whether the gene-environment equivalence assumption is reasonable. Use causal language carefully, clarifying that IV estimates may provide causal effects only under certain assumptions	13	Paragraph starting from "PZP is a protein initially described as a major pregnancy-associated protein, showing elevated levels during pregnancy and higher expression levels in females than in males"
		c) Clinical relevance: Discuss whether the results have clinical or public policy relevance, and to what extent they inform effect sizes of possible interventions	13	Paragraph starting from "PZP is a protein initially described as a major pregnancy-associated protein, showing elevated levels during pregnancy and higher expression levels in females than in males"
17	**Generalizability**	Discuss the generalizability of the study results (a) to other populations, (b) across other exposure periods/timings, and (c) across other levels of exposure	14-15	The SD-pQTLs and their associations with health disorders also require careful consideration, as the analysis was limited to Caucasian individuals from the UK Biobank, leveraging its large sample size. Additionally, disease prevalence differs between males and females, as observed in our dataset.<br>Another limitation of our MR analysis is that it was based on individuals of Caucasian ancestry from the UK Biobank, as SD-pQTLs from other ancestries would have limited power in an MR framework. Restricting MR analyses to ancestrally homogenous samples reduces the risk of population stratification that can lead to violation of the independence and exclusion restriction MR assumptions [46]. However, transferring MR results across ancestries is challenging due to differences in LD patterns and allele frequencies [46], although methods to facilitate trans-ancestry MR have been proposed [47]. It should also be noted that aside from population stratification, MR analyses can be biased by assortative mating, dynastic events (the direct effect of one's parents on a phenotype), or selection bias, such as participation bias in the UK Biobank. Such processes can confound the relationship

				between SD-pQTLs and disease outcomes, potentially violating the MR independence assumption [48]
	**OTHER INFORMATION**			
18	**Funding**	Describe sources of funding and the role of funders in the present study and, if applicable, sources of funding for the databases and original study or studies on which the present study is based	23	The Funding section
19	**Data and data sharing**	Provide the data used to perform all analyses or report where and how the data can be accessed, and reference these sources in the article. Provide the statistical code needed to reproduce the results in the article, or report whether the code is publicly accessible and if so, where	23	The Availability of data and materials section
20	**Conflicts of Interest**	All authors should declare all potential conflicts of interest	23	The Competing interests section

This checklist is copyrighted by the Equator Network under the Creative Commons Attribution 3.0 Unported (CC BY 3.0) license.

1. Skrivankova VW, Richmond RC, Woolf BAR, Yarmolinsky J, Davies NM, Swanson SA, et al. Strengthening the Reporting of Observational Studies in Epidemiology using Mendelian Randomization (STROBE-MR) Statement. JAMA. 2021;under review.

2. Skrivankova VW, Richmond RC, Woolf BAR, Davies NM, Swanson SA, VanderWeele TJ, et al. Strengthening the Reporting of Observational Studies in Epidemiology using Mendelian Randomisation (STROBE-MR): Explanation and Elaboration. BMJ. 2021;375:n2233.
